# Is Routine Neuroimaging Needed in Adult-Onset Isolated Cervical Dystonia?

**DOI:** 10.5334/tohm.1049

**Published:** 2025-08-06

**Authors:** Elina Myller, Oskari Korhonen, Juho Joutsa

**Affiliations:** 1 Turku Brain and Mind Center, Clinical Neurosciences, University of Turku, Turku, Finland; 2 Neurocenter, Turku University Hospital, Turku, Finland

**Keywords:** Cervical dystonia, neuroimaging

## Abstract

**Background:**

Clinical practices regarding neuroimaging in isolated cervical dystonia vary across countries and there are no published studies investigating the need of routine neuroimaging in this patient population.

**Objectives:**

To investigate if structural neuroimaging is needed in patients with isolated cervical dystonia.

**Methods:**

Patients with adult-onset cervical dystonia were identified from a systematic search of the medical records of Turku University Hospital 1996–2022. Clinical and structural neuroimaging data were reviewed by the investigators to evaluate the etiology of dystonia, specifically to identify cases of secondary dystonia caused by structural brain abnormalities.

**Results:**

365 patients with cervical dystonia without other movement disorders with presumed idiopathic or uncertain etiology prior to brain imaging were identified. 282 (77.3%) were scanned using head MRI or CT. Acquired brain lesions were identified in nine (2.5% of all patients) and were significantly more common in patients with vs. without (i.e. isolated) other neurological features (*P* < 0.001). Lesions in patients with other neurological features were considered likely (n = 4) or possibly (n = 2) causal, but all lesions in patients with isolated cervical dystonia (n = 3) were considered incidental. None of the patients showed signs of progressive neurodegeneration.

**Conclusions:**

Routine neuroimaging is not necessary in patients with adult-onset isolated cervical dystonia.

**Highlights:**

Studies investigating the need of structural neuroimaging in isolated, adult-onset cervical dystonia are scarce and opinions on this issue are divided among experts.

In this study, we reviewed clinical and imaging data of all patients with cervical dystonia with presumed idiopathic or uncertain etiology prior to brain imaging treated at a regional tertiary care hospital between 1996–2022 to investigate the yield of structural brain imaging in these patients.

Of the included 365 patients, none showed evidence of progressive neurodegeneration underlying the symptoms and only six (1.6%) showed acquired brain lesions that were considered possibly or likely causal for cervical dystonia.

All the six patients with possible or likely lesion-induced cervical dystonia showed cervical dystonia combined with other neurological features, indicating that routine neuroimaging is not needed in isolated, adult-onset cervical dystonia.

## Introduction

Cervical dystonia is by far the most common form of adult-onset focal dystonia [1], characterized by sustained or intermittent neck movements caused by involuntary muscle contractions resulting in abnormal movements and postures of head, neck, and/or shoulders [2]. Cervical dystonia is classified as isolated when it’s not associated with other movement disorders (except for tremor), neurological or systemic features [3]. The etiology of adult-onset cervical dystonia is most often idiopathic (i.e. unknown genetic or acquired causes where there is no evidence of neuroanatomical lesions) [2,3]. However, it can also be associated with neuroanatomical lesions [3], commonly localizing to the cerebellum or basal ganglia [4,5].

According to the recent consensus statement of the International Parkinson and Movement Disorder Society (MDS) Dystonia Study Group, the diagnosis of cervical dystonia is clinical [2]. Although the panel felt that routine neuroimaging is not necessary in isolated cervical dystonia without atypical features, it noted lack of evidence and variable practices across countries with some experts advocating for routine neuroimaging [2,6]. Thus, the panel’s conclusion was that currently there is no consensus regarding the need for routine neuroimaging in the diagnostic workup in isolated cervical dystonia [2].

The aim of this study was to investigate if routine neuroimaging is needed in the diagnostic workup of isolated cervical dystonia. To this end, we reviewed the clinical records and neuroimaging findings of patients with adult-onset cervical dystonia with presumed idiopathic or uncertain etiology prior to brain imaging treated between 1996–2022 at Turku University Hospital, the regional tertiary care hospital of Southwest Finland.

## Methods

### Patient selection

In this retrospective cohort study, all adult patients (age 18 years or above at the onset of symptoms) with ICD-10 code G24.3 (cervical dystonia) treated at the neurology clinic at Turku University Hospital from the inception of the electronic medical records and use of ICD-10 in 1996 to January 2022 were identified using a systematic search. The search identified 761 patients, whose medical records were reviewed in detail by the authors (E.M. & O.K. in consultation with J.J.).

**Figure 1:**
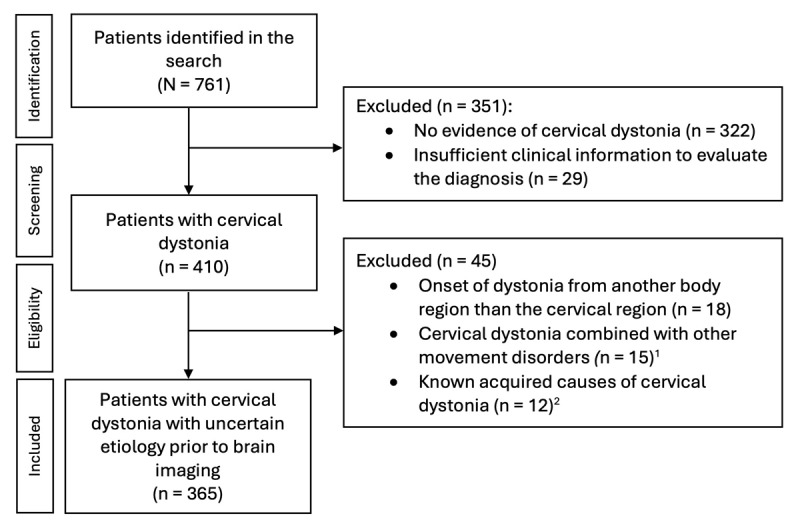
Systematic search process for patients with cervical dystonia. ^1^Parkinson’s disease n = 9, ataxia n = 3, progressive supranuclear palsy n = 2, corticobasal syndrome n = 1. ^2^Drug-induced cervical dystonia n = 8, developmental disability due to infectious brain injury in the childhood with delayed-onset cervical dystonia in the adulthood n = 1, dystonic cerebral palsy with delayed-onset dystonia in the adulthood n = 1, developmental disability due to chromosomal aberration, deformation of the spinal column and delayed-onset cervical dystonia in the adulthood n = 1, brain injury due to Arnold Chiari malformation, hydrocephalus, cerebellar tonsillectomy and posterior fossa decompression surgery prior to the onset of cervical dystonia n = 1. Of note, none of these patients had isolated cervical dystonia. None of patients met the diagnostic criteria of genetic cervical dystonia.

The case selection flow chart is presented in Figure 1. Patients with no evidence of cervical dystonia and patients with insufficient or inconsistent clinical information to confirm the diagnosis of cervical dystonia were excluded [2]. In addition, patients with other dystonia types later spreading to cervical region, patients with dystonia combined with other movement disorders, and patients with known genetic or acquired etiology other than lesions (e.g. drug-induced dystonia) were excluded to limit the analyses to patients with cervical dystonia with presumed idiopathic or uncertain etiology prior to brain imaging at the time of diagnostic evaluation.

The study protocol was approved by the Hospital District of Southwest Finland and the need for separate ethical committee evaluation or patient consent were waived because of the retrospective nature of the study, according to the institutional and national rules and regulations. The study was conducted according to the principles of the Declaration of Helsinki.

### Data analysis

Of the included patients, demographic and clinical features were extracted from the medical records and evaluated based on the most recent consensus statements [2,3,7]. The clinical information included sex, age at diagnosis, family history of cervical dystonia, presence of an effective sensory trick, pain related to cervical dystonia and tremor at the time of diagnosis. In addition, other neurological features at the onset of dystonia were documented [3].

All head CT and MRI scans had been initially evaluated by a radiologist and were re-evaluated by the authors to identify intracranial lesions (acquired lesions or non-progressive neurodevelopmental anomalies) and evidence for progressive neurodegeneration [3].

As there are no established guidelines for the diagnosis of lesion-induced movement disorders including cervical dystonia [8], the likelihood of the causality of lesions identified in brain imaging were evaluated case-by-case as likely, possible and unlikely. Patients with (sub)acute or short delay (<6 mo) symptom onset and a commonly reported causal lesion location were considered as likely causal [5]. Lesions not meeting the criteria for likely causal but occurring in a location previously reported to cause dystonia with a latency less than the maximum latency in lesion-induced dystonia reported in the literature (5.5 years) were considered as possibly causal [5]. Lesions that did not meet either of these criteria were considered unlikely to be causal (classified as incidental).

### Statistical analyses

All statistical analyses were performed using IBM SPSS Statistics (version 29, IBM Corp., New York, USA). The assumption of normality was evaluated visually from histograms, together with Shapiro-Wilk tests. Demographic and clinical features between patients with and without other neurological features at the onset of dystonia were tested using Mann-Whitney U test or Fisher Exact test, as appropriate. *P*-values < 0.05 were considered significant.

## Results

### Demographic and clinical characteristics

Demographic and clinical characteristics of the 365 patients with cervical dystonia with presumed idiopathic or uncertain etiology prior to brain imaging is shown in Table 1. 282 (77.3%) of the patients were scanned using CT or MRI with 148 (52.5%) of these specifically because of cervical dystonia based on the imaging referral. The remaining 134 patients (47.5%) were scanned with heterogenous indications unrelated to cervical dystonia (most commonly acute/subacute neurological issues without any temporal association to dystonia, such as later stroke symptoms, head traumas, or headache). Fourteen out of the 365 patients (3.8%) showed other neurological features at the onset of cervical dystonia (Table 1).

**Table 1:** Demographic and clinical characteristics.


	ALL PATIENTS n = 365	NO OTHER NEUROLOGICAL FEATURES (I.E. ISOLATED) n = 351	OTHER NEUROLOGICAL FEATURES n = 14	*P*-VALUE

**Demographic features**				

Age at diagnosis, median (range)	49.0 (20–92)	49.0 (20–92)	56.0 (27–75)	0.477

Sex (F), *n* (%)	285 (78.1%)	273 (77.8%)	12 (85.7%)	0.743

Family history of cervical dystonia, *n* (%)	279 (7.9%)	27 (7.7%)	2 (14.3%)	0.307

Duration of follow-up in months, median (range)	115 (1–313)	115 (1–313)	94.5 (4–313)	0.638

**Clinical features of dystonia**				

Effective sensory trick, *n* (%)	44 (12.1%)	43 (12.3%)	1 (7.1%)	1.000

Pain related to cervical dystonia, *n* (%)	240 (65.8%)	233 (66.4%)	7 (50.0%)	0.252

Head/neck tremor, *n* (%)	234 (64.1%)	224 (63.8%)	10 (71.4%)	0.560

Upper limb tremor, *n* (%)	33 (9.0%)	31 (8.8%)	2 (14.3%)	0.367

**Brain imaging**				

Brain imaging, *n* (%)	282 (77.3%)	268 (76.4%)	14 (100%)	**0.046**

CT/MRI, *n*	37/245	37/231	0/14	0.228

Acquired brain lesions^1^, *n (*%)	9/282 (3.2%)	3/268 (1.1%)	6/14 (42.9%)	**<0.001**

Lesion-induced cervical dystonia^2^, n (%)	6/282 (2.1%)	0/268 (0.0%)	6/14 (42.9%)	**<0.001**


Patients with cervical dystonia with presumed idiopathic or uncertain etiology prior to brain imaging with and without other neurological features at the onset of dystonia. ^1^All acquired brain lesions before the onset of cervical dystonia. ^2^Likely (n = 4) or possible (n = 2) lesion-induced cervical dystonia (see Supplementary table 1 for more details).

Of patients who had undergone structural brain imaging, the findings were unremarkable in 264 patients (93.6%). None of patients had evidence of progressive neurodegeneration and nine (3.2% of scanned; 2.5% of all patients) had benign incidental findings, such as small cysts and uncomplicated venous anomalies. Nine patients (3.2%; 2.5%) showed acquired brain lesions that had occurred before the onset of cervical dystonia (Table 1). Of these nine patients, the lesions were interpreted to be likely causal in four, possibly causal in two, and incidental in three patients (Supplementary table 1). The etiology of cervical dystonia was interpreted to be idiopathic in all patients without brain lesions, as well as in patients with incidental lesions.

### Isolated cervical dystonia vs. cervical dystonia combined with other neurological features

There were no significant differences in the demographic or clinical features of dystonia at the time of diagnosis between patients with and without (i.e. isolated dystonia) other neurological features (Table 1). As expected, use of structural brain imaging and acquired brain lesions were significantly more common in patients with compared to without other neurological features (*P* < 0.05, Table 1). Lesions in patients with other neurological features (n = 6) were interpreted to be likely (n = 4) or possibly (n = 2) causal for cervical dystonia (Supplementary table 1). In contrast, none of patients without other neurological features (isolated dystonia, n = 3) had likely or possibly causal lesions (Supplementary table 1). Possible/likely lesion-induced etiology was significantly more common in patients with vs. without other neurological features (*P* < 0.001, Table 1).

## Discussion

The aim of this study was to investigate if routine structural brain imaging is needed in the diagnostic workup in patients with adult-onset isolated cervical dystonia. Our results showed that none of patients with isolated cervical dystonia had evidence for neuroanatomical brain lesions (including evidence of progressive neurodegeneration) that could be considered likely or possibly causal for dystonia. The results are consistent with the views of the MDS Dystonia Study Group panel and provide evidence for not using routine structural brain imaging in this patient population [2]. In contrast, cervical dystonia with associated or atypical features warrants brain imaging [2,3], as demonstrated by a high rate of acquired causal brain lesions in patients with other neurological features in the present study.

Overall, the rate of abnormal imaging findings (benign incidental or acquired) detected in the scans in the entire sample was small (6.4%), roughly aligning with rate of incidental findings in neuroimaging studies with healthy volunteers (3.7–9.4%) [9,10,11]. Structural brain imaging provided novel information about the etiology of dystonia in only six patients (1.6%), all combined with other neurological features. To our knowledge, there are no prior studies investigating the need for routine neuroimaging specifically in patients with presumed idiopathic or uncertain etiology prior to neuroimaging. However, our findings are supported by a prior Italian study that did not find causal brain lesions from patients with diagnosis of idiopathic cervical dystonia [6]. However, this study excluded patients with known lesion-induced cervical dystonia and, therefore, would not have captured cases identified as such early in the diagnostic workup.

This study has some limitations that should be acknowledged. First, due to the retrospective study design, the diagnoses could not be confirmed with a clinical examination by a movement disorder neurologist. However, all cases were collected from a regional university hospital and had been evaluated by a neurologist, and the clinical notes of the cases were carefully re-evaluated by the investigators to ensure that the clinical descriptions were consistent with cervical dystonia, making incorrect diagnoses unlikely. Second, the rates of individual clinical features should be interpreted with caution, as these are not systematically investigated and documented in everyday clinical practice. Finally, as there are no established guidelines for the diagnosis of lesion-induced cervical dystonia [8], the evaluation of the causality of lesions was based on the anatomical lesion location and temporal relationship, evaluated based on available information of cases of lesion-induced dystonia reported in the literature [5]. For example, patients with a very long time interval between the lesion and onset of dystonia, exceeding the longest latency ever reported for lesion-induced dystonia, were considered as unlikely to have lesion-induced dystonia. However, the role of lesions, which did not meet our criteria for likely or possibly causal lesions, on the pathogenesis of dystonia cannot be definitely excluded [3].

To conclude, our results demonstrate that the yield of clinical structural neuroimaging in patients with isolated cervical dystonia is minimal, as none of patients without other neurological features had etiologically relevant imaging findings. Our findings provide evidence supporting not using routine neuroimaging in patients with adult-onset isolated cervical dystonia in the diagnostic workup.

## Data Accessibility Statement

The authors confirm that data supporting the findings of this study are available within the article and in the Supplementary material. Additional data supporting the findings are not shared.

## Additional File

The additional file for this article can be found as follows:

10.5334/tohm.1049.s1 Supplementary Table 1. Patients with cervical dystonia and acquired brain lesions preceding the onset of dystonia.
