# Supplementary material for: Is Routine Neuroimaging Needed in Adult-Onset Isolated Cervical Dystonia?
Source: Tremor Other Hyperkinet Mov (N Y). 2025 Aug 6;15:36. doi: 10.5334/tohm.1049 (PMC12330805; doi:10.5334/tohm.1049)
Supplement: Supplementary Table 1. — Patients with cervical dystonia and acquired brain lesions preceding the onset of dystonia. [file tohm-15-1-1049-s1.pdf]

Supplementary Table 1. Patients with cervical dystonia and acquired brain lesions preceding the onset of dystonia.

Patient	Lesion etiology	Number of lesions	Lesion locations	Latency	Other neurological features at the onset of dystonia	Lesion-induced
1	Ischemic stroke	2	Cerebellum R, thalamus R	2 weeks	Dysarthria, sensory deficit L, ataxia L, balance impairment	Likely
2	Ischemic stroke	2	Insular cortex R, parietal white matter L	Acute	Upper limb motor paresis L, lower limb motor paresis R, dysphagia, acute-onset cognitive defect	Likely
3	Ischemic stroke	1	Cerebellum L	< 3 months	Ataxia, dysmetria and upper limb sensory deficit L	Likely
4	Traumatic brain injury	1	Temporal cortex L	Acute	Upper limb numbness and paresthesia R, constant intense headache, balance impairment	Likely
5	Multiple sclerosis	> 20	Juxtacortical, periventricular, infratentorial, R+L	3,5 years	Right-predominant lower limb sensorimotor paresis R+L, upper + lower limb ataxia R+L, balance impairment	Possible
6	Multiple sclerosis	10	Juxtacortical, periventricular, infratentorial, R+L	4 years	Lower limb sensory deficit L	Possible
7	Ischemic stroke	1	Internal capsule L	N.a.	None[1]	Unlikely
8	Ischemic stroke	1	Cerebellum L	25 years	None[1]	Unlikely
9	Ischemic stroke	4	Frontal white matter R, cerebellum L, precentral gyrus L, occipital cortex L	9, 18 and 24 years	None[1]	Unlikely

The patients with brain lesions included 3 males and 6 females with mean (sd, range) age 56.8 (15.9, 27-80) years. N.a. = not available. R = right. L = left. [1]The stroke lesion for patient 7 was asymptomatic, whereas patients 8 and 9 were asymptomatic from the infarctions at the onset of dystonia.
